# Artificial Intelligence in the Evolution of Customized Skincare Regimens

**DOI:** 10.7759/cureus.82510

**Published:** 2025-04-18

**Authors:** Mary Grace Hash, Alyssa Forsyth, Bret-Ashleigh Coleman, Vivian Li, Julia Vinagolu-Baur, Kelly M Frasier

**Affiliations:** 1 Dermatology, Edward Via College of Osteopathic Medicine, Auburn, USA; 2 Dermatology, Texas College of Osteopathic Medicine, Fort Worth, USA; 3 Medical School, Edward Via College of Osteopathic Medicine, Auburn, USA; 4 Medicine, Nuvance Health, Vassar Brothers Medical Center, Poughkeepsie, USA; 5 Medical Education, State University of New York Upstate Medical University, Syracuse, USA; 6 Dermatology, Northwell Health, New Hyde Park, USA

**Keywords:** ai-based therapy, artificial inteligence, artificial intelligence in medicine, multifunctional skincare, sensitive skin

## Abstract

Artificial intelligence (AI) is revolutionizing multiple industries, including health care and cosmetics, with particular promise in personalized skincare. This literature review explores recent advancements in AI technology that enable the development of highly customized skincare regimens. By integrating intrinsic factors such as skin type, pigmentation, texture, and genetic predispositions with external influences such as product ingredients, lifestyle choices, and environmental conditions, AI offers a sophisticated approach to managing skin health. AI’s ability to analyze and synthesize vast amounts of data allows for a more comprehensive understanding of how these factors interact, facilitating the prediction of long-term skin health outcomes with greater accuracy. Moreover, AI-driven innovations enable the creation of dynamic, adaptive skincare routines that adjust in real-time to physiological changes and external conditions, ensuring a personalized treatment that evolves with each individual’s dermatological needs. As AI technology advances, its role in skincare is expected to deliver increasingly personalized and effective therapies, shifting from traditional, static skincare approaches to a more dynamic paradigm. This transformation holds great promise for improving dermatological outcomes and enhancing patient satisfaction through tailored real-time skincare management.

## Introduction and background

Artificial intelligence (AI) is emerging as a transformative force across numerous fields, with healthcare at the forefront of this technological revolution. By analyzing vast amounts of data using advanced algorithms, AI has improved personalized care, leading to more accurate diagnoses, optimized treatments, and better patient management [1]. In dermatology, AI has demonstrated measurable success in enhancing the accuracy of melanoma detection through dermoscopic image analysis, automating histopathological classification of basal cell carcinoma subtypes, and evaluating post-procedural outcomes in laser resurfacing and injectables by quantifying skin texture and symmetry changes [2]. This trend is extending into the skincare industry, where personalized solutions are essential for achieving optimal results. Traditional skincare approaches frequently rely on generalized formulations and recommendations that overlook the intersection between individual skin physiology, such as barrier function and sebum production, and contextual variables like climate, pollution exposure, and cultural skincare practices, ultimately limiting their effectiveness across diverse populations. AI offers tailored recommendations that enhance patient satisfaction and outcomes through customized skincare regimens [3]. As AI-driven tools become more prevalent in dermatology, they are set to transform the field by providing precise, adaptive, and personalized skincare solutions tailored to each individual.

The integration of AI in skincare addresses the complex interplay of intrinsic and extrinsic factors influencing skin health. Intrinsic elements such as genetics, age, and skin type interact with extrinsic factors like environment and lifestyle to affect skin condition [4]. The traditional trial-and-error approach to skincare, where consumers experiment with various products without personalized guidance, often leads to frustration and suboptimal results [5]. Understanding the intricate relationship between skin biology and external elements is vital for success, making personalized skincare regimens increasingly essential. AI presents a promising solution to these challenges by integrating data from multiple sources to create skincare routines that adjust to internal and external changes. Recent advancements in AI, such as machine learning, deep learning, and computer vision, have transformed healthcare diagnostics, leading to more precise and individualized treatment plans [6,7]. These technologies are now being applied to skincare, allowing AI to analyze individual skin profiles, predict outcomes, and recommend real-time adjustments to skincare routines [8]. This shift signifies a paradigm change from static, generalized skincare approaches to a dynamic model that evolves according to the user’s skin needs.

This review explores the integration of AI into skincare personalization, focusing on its ability to overcome the limitations of traditional approaches. By offering customized and adaptive solutions, AI holds the potential to enhance skin health and increase patient satisfaction, ushering in a new era of personalized skincare that is more effective and aligned with individual characteristics and needs. Additionally, as AI technology advances, the capacity to analyze vast dermatological data will result in even more accurate recommendations. This development provides consumers with personalized skincare routines and promotes a deeper understanding of skin health among diverse populations.

## Review

The need for personalized skincare

The variability of human skin due to genetics, age, hormonal changes, lifestyle habits, and environmental exposures underscores the limitations of traditional skincare products. Conventional products are typically designed for broad skin categories, such as dry or oily, but fail to accommodate each individual's unique and evolving needs. Skin conditions fluctuate due to intrinsic factors like hormonal changes and aging, as well as extrinsic factors such as pollution and climate variations. AI provides a solution by synthesizing individual skin profiles with real-time data, creating dynamic skincare regimens that adjust to each user's specific needs. This comprehensive approach addresses individuals’ unique requirements in ways that traditional one-size-fits-all products cannot. Moreover, AI-driven models synthesize multiple data sources, including user inputs, dermatological studies, and imaging tools, to create highly customized skincare regimens [6,7]. For instance, AI-driven skincare applications can continuously monitor and analyze changes in factors like hydration, sensitivity, and acne patterns, dynamically refining product recommendations to better match the skin’s evolving needs, leading to more effective and individualized care [9]. As AI technology continues to advance, its ability to provide highly adaptive, personalized skincare has the potential to revolutionize the industry by addressing each individual's specific, changing needs in real-time. Thus, AI is not only meeting but exceeding the demands of modern skincare by offering targeted and adaptable solutions that static methods fail to deliver.

AI-powered skincare tools

AI is revolutionizing personalized skincare by enabling companies to deliver highly customized beauty solutions. The landscape of AI-powered personalized product recommendations is rapidly evolving. Early AI platforms primarily relied on questionnaires, asking users about their skin type and dominant concerns to create a basic personalized profile [6,7]. While these platforms provided some level of customization, their effectiveness was limited by reliance on subjective self-assessments.

Recent advancements in AI and cosmetic dermatology have enabled more objective, data-driven approaches. For example, new technologies now utilize photo analysis to customize skincare regimens based on visual assessment. L’Oréal, after acquiring Modiface, a company specializing in augmented reality and AI, has positioned itself at the forefront of this transformation. The company’s “SkinConsult AI” leverages deep learning algorithms trained on millions of facial images to evaluate skin conditions across multiple dimensions, such as wrinkles, texture, and pigmentation [10]. Users can upload selfies, enabling the AI to analyze their skin and recommend products tailored to their concerns. Additionally, the L’Oréal Perso device exemplifies this capability by using ModiFace technology to analyze user-uploaded facial photographs. This tool detects skin concerns such as enlarged pores and fine lines, while also factoring in environmental data like weather conditions and ultraviolet (UV) index, along with user preferences regarding treatment and skincare goals. Consequently, Perso can create tailored products on demand, including skincare items, foundation, and lipstick [10,11]. This innovative and adaptable strategy demonstrates L’Oréal’s commitment to harnessing AI in creating highly individualized skincare products, setting a new standard for personalized beauty solutions. These technologies continue to evolve, incorporating more diverse data to offer increasingly personalized skincare recommendations.

Proven Skincare is another pioneer in AI-driven skincare, utilizing the “Skin Genome Project” to develop hyper-personalized formulations through extensive data analysis. The project evaluates over 20,000 ingredients, 100,000 products, millions of customer testimonials, and thousands of peer-reviewed scientific articles [12]. Users begin their journey by completing an AI-assisted questionnaire that collects detailed information about their skin type, medical history, stress levels, dietary habits, and environmental exposures. This comprehensive data allows Proven’s AI to propose tailored product formulations, continuously refining them based on user feedback and changing skin conditions. This dynamic adaptability addresses immediate skincare needs and supports long-term skin health by responding to physiological changes, such as hormonal fluctuations and seasonal variations.

Beyond customization, the role of AI in skincare extends to enhancing real-time skin monitoring. AI-powered applications utilize computer vision and facial recognition technologies to evaluate essential skin characteristics, such as texture, pigmentation, hydration, and blemishes. For example, a deep learning model developed by Yoon et al. effectively identifies skin morphology and enhances the characterization of features like pores and wrinkles [13]. Seité et al. developed an AI algorithm trained on a dataset comprising thousands of facial images to assess acne severity using the Global Evaluation of Acne (GEA) scale. The model achieved a 68% agreement rate with dermatologist evaluations, a figure that approximates the level of inter-rater concordance typically observed among dermatologists themselves. This finding not only supports the algorithm’s potential as a clinical decision-support tool but also underscores the inherent subjectivity and variability in human grading of acne severity, emphasizing the need for standardized, reproducible assessment methods in dermatologic practice [9]. These capabilities underscore the importance of AI not only in personalizing skincare regimens but also in monitoring skin health alongside professional dermatological care.

In addition, platforms like Haut.AI use machine learning algorithms to assess user-submitted images and provide personalized skincare recommendations based on real-time skin data [14]. This technology combines inputs from dermatological research and user-provided data to create customized skincare regimens. These innovations have received positive feedback, with users reporting improved skincare results and greater satisfaction with products designed to meet their specific needs. 

AI in product and ingredient analysis 

AI plays a crucial role in personalized skincare by analyzing product ingredients and assessing their effects on various skin types and conditions. AI algorithms analyze the chemical composition of skincare products by parsing ingredient lists for compounds such as retinoids, alpha-hydroxy acids, parabens, and sulfates, then cross-reference these with individual skin profiles that include sensitivity markers, allergy history, and conditions like rosacea or eczema. For instance, the algorithm may flag benzoyl peroxide as potentially irritating for a user with compromised barrier function or recommend niacinamide for someone with hyperpigmentation and oily skin, enabling more tailored and clinically relevant product recommendations [15]. By utilizing comprehensive ingredient databases and dermatological research, AI tools provide a level of precision that exceeds manual analysis, enabling consumers to align their skincare regimen with their specific skin concerns and sensitivities. Moreover, AI-driven models predict critical parameters like the structure-property relationships of surfactants, polymers, and preservatives in cosmetics, which are essential for ensuring product stability and efficacy [15]. These advancements in AI have streamlined the formulation process, reducing trial-and-error inefficiencies and enhancing the personalization of skincare solutions.

AI systems also excel at detecting harmful components, such as potential allergens or irritants, helping users avoid ingredients that may exacerbate their skin conditions, including preservatives, fragrances, or harsh exfoliants. A study by Kalicińska et al. evaluated an in silico system that modeled the sensitizing potential of cosmetic product ingredients based on their molecular properties, allowing for ethical predictions without the need for animal testing or in vitro methods [16]. This highlights the effectiveness of AI’s ingredient analysis in predicting potential immune responses. In the context of personalized skincare, a similar model could incorporate knowledge of individual sensitivities and known allergens to compile a list of ingredients to avoid based on their molecular structures. This technology has the potential to mitigate adverse reactions, such as pruritus and inflammation, thereby improving skincare regimens.

AI platforms, such as ingredient scanners and skincare diagnostic tools, provide personalized insights by evaluating how product ingredients interact with a user’s specific skin needs. These platforms analyze the active components in skincare products, assessing their effectiveness in addressing issues such as acne, hyperpigmentation, aging, or sensitivity. For instance, an AI-driven tool might recommend ingredients like niacinamide to reduce hyperpigmentation or hyaluronic acid to enhance hydration based on an individual’s skin analysis. One system developed by Lee et al. integrates data from skin-analyzing facial scans and product ingredient analysis to create personalized recommendations tailored to an individual’s skincare needs [17]. Unlike Perso, this system focuses on analyzing the ingredients present in cosmetic products currently available on the market, ensuring that users select skincare ingredients optimized for their particular skin type and aesthetic goals [17]. Such systems enhance the precision of ingredient recommendations, effectively addressing diverse skin needs.

Dynamic and predictive skincare solutions

AI has redefined skincare by enabling both real-time adjustments and predictive modeling to address immediate and long-term skin health needs. AI-driven skincare platforms, such as L’Oréal’s Perso and Shiseido’s Optune, integrate real-time environmental data-including UV index, particulate matter (PM2.5) levels, humidity, and ambient temperature, sourced from geolocation APIs and weather databases. These platforms dynamically adjust product recommendations, such as increasing SPF strength and antioxidant serums during high UV and pollution days or suggesting humectant-rich moisturizers during low-humidity, cold conditions, to optimize skin barrier protection and hydration in response to shifting environmental stressors [18-21]. For example, on days with high UV exposure, AI might suggest products with higher sun protection factor (SPF) protection, antioxidants, or soothing ingredients like aloe vera. Environmental factors play a crucial role in skin health, impacting hydration levels, barrier function, and susceptibility to irritation. Fluctuations in humidity can either increase sebum production or accelerate moisture loss, contingent upon whether humidity levels are elevated or diminished [22-24]. In humid conditions, AI systems might recommend lighter, oil-free products to minimize excess oil, while recommending richer, occlusive moisturizers when the air is dry and cold. By continuously adapting to these external conditions, AI-driven skincare regimens offer a more comprehensive and protective approach compared to static routines, optimizing product effectiveness and user satisfaction.

In addition to environmental factors, AI addresses real-time physiological changes in the skin. This is particularly pertinent to skin conditions that fluctuate due to hormonal cycles, stress levels, hydration status, and lifestyle influences. AI-driven skincare systems often incorporate data from wearable skin sensors or smart devices that measure physiological parameters such as moisture levels, skin pH, and transepidermal water loss (TEWL) [25]. For instance, when an increase in TEWL is detected, indicating a compromised barrier function, AI algorithms may recommend more occlusive formulations to enhance moisture retention and repair the skin barrier. This proactive methodology not only improves treatment efficacy but also mitigates the risk of adverse reactions by adapting to the skin’s immediate requirements. Consequently, AI-driven skincare regimens foster healthier skin by responding to the user’s physiological changes in real-time rather than adhering to predetermined routines that may not align with evolving conditions. 

AI’s capability extends beyond real-time adjustments to predictive modeling, offering significant benefits for addressing long-term skin needs and preventing future issues. By analyzing genetic predispositions, environmental conditions, and past product efficacy, AI can forecast how specific regimens will affect an individual's skin over time. For instance, emerging AI systems are beginning to integrate genetic markers, such as polymorphisms associated with collagen degradation, inflammation, or sebum production, with environmental exposures like UV radiation, pollution, and humidity. While still in the early stages, this approach holds the potential to enhance risk stratification for conditions such as premature wrinkling, photoaging, and acne susceptibility, informing more personalized prevention strategies rather than definitive predictions [26,27]. This predictive ability enables long-term prevention strategies while addressing immediate concerns, resulting in more effective outcomes, particularly for chronic skin conditions such as acne. For example, individuals prone to hyperpigmentation may be guided toward ingredients like vitamin C while avoiding irritants [28]. This predictive modeling is beneficial for chronic skin issues, as AI systems adapt recommendations based on real-time data, such as sebum levels or hydration needs, ensuring skincare remains effective and responsive [3,29]. This predictive adaptability empowers individuals to manage their skin health proactively and effectively over time.

Recent advancements in AI have also enhanced our understanding of chronic skin conditions such as acne. A study by Li et al. examined the severity of acne among over one million adult Chinese women using an AI algorithm to analyze high-resolution smartphone images. The findings highlight that although acne severity diminishes after age 25, it gradually increases after reaching its lowest point between ages 40 and 44. Contributing factors such as oily skin, makeup use, poor diet, urban living, and climate conditions were found to correlate with increased acne severity [26]. Insights from this research can guide personalized skincare and public health strategies, emphasizing the role of AI in identifying trends and addressing disparities in skin health.

Furthermore, AI systems can continuously track changes in skin conditions by analyzing user-submitted photographs, providing real-time feedback and adaptive skincare recommendations. For instance, an individual may use an AI application to assess daily hydration levels, receiving alerts when their skin shows excessive dryness [30]. Research has demonstrated the accuracy of machine learning models, including random forest algorithms, in detecting hydration levels. As more advanced techniques, such as neural networks, develop, these tools are expected to enhance the precision of personalized skincare. Over time, AI systems learn from individual responses to refine future recommendations, ensuring skincare regimens are tailored to the user’s unique chemistry and environmental factors. This feature enables AI to enhance skincare routines in real-time, ensuring effectiveness even with changes in external conditions or individual skin requirements. Additionally, AI evaluates how a person’s skin reacts to specific products, storing these responses to provide increasingly personalized suggestions in the future [3,29]. With repeated use, AI can recommend skincare ingredients specifically suited to an individual’s unique chemistry and environmental factors, maximizing the safety and efficacy of skincare regimens. 

Benefits and limitations of AI in skincare personalization 

One of the primary advantages of AI in skincare personalization is its ability to offer data-driven precision and dynamic real-time adjustments. As mentioned previously, AI can process vast amounts of data, analyzing factors such as an individual’s skin type, age, genetics, lifestyle, and external influences like UV exposure, pollution, and climate. This multidimensional approach allows AI to provide highly individualized and adaptive skincare recommendations that evolve alongside the user’s changing skin needs. For instance, AI platforms like Proven Skincare’s system can suggest specific ingredients, such as antioxidants for those exposed to high pollution or retinoids for users aiming to reduce signs of aging, by considering dozens of variables to create customized formulations [3,6,31]. This adaptability reduces the guesswork typically involved in switching products based on trial and error, making it easier for users to maintain suitable skincare routines. By continuously updating product recommendations in response to factors like aging, hormonal fluctuations, or environmental shifts, AI ensures more effective, responsive, and targeted treatments than traditional one-size-fits-all approaches.

AI also plays a significant role in making skincare advice more accessible. Through mobile apps and online platforms, AI-powered tools offer personalized skin health assessments and customized product recommendations, eliminating the need for in-person dermatological consultations [6]. This democratization of expert skincare advice is particularly beneficial for those living in remote areas or individuals who may not have the financial means to consult with dermatologists regularly. Apps like L’Oreal’s SkinConsult AI and Skinsei allow users to upload photos, receive skin analyses, and obtain tailored skincare recommendations from the comfort of their own homes. While these platforms increase access to personalized skincare, the cost of AI-driven personalized products may still be prohibitive for some, particularly in lower socioeconomic groups, creating a gap between those who can afford premium skincare solutions and those who cannot.

However, the widespread use of AI in skincare raises concerns regarding data privacy and security. AI-based systems often require users to share sensitive personal data, including images of their skin, genetic information, and details about their lifestyle [32,33]. The collection, analysis, and storage of sensitive data, ranging from facial imagery and biometric markers to genetic profiles and dermatologic histories, in healthcare and cosmetic AI applications pose significant privacy risks. These datasets are often stored on cloud-based platforms or shared with third-party service providers, increasing vulnerability to cyberattacks, data leaks, or unauthorized access. In healthcare settings, breaches could expose confidential medical information protected under regulations such as HIPAA, potentially leading to discrimination, stigmatization, or insurance-related consequences. In the cosmetic domain, where regulatory oversight is often less stringent, user data may be exploited for targeted marketing or sold to advertisers without explicit consent. Moreover, the integration of AI with wearable skin sensors and mobile health apps raises further concerns about continuous data tracking and potential misuse of behavioral and locational metadata, highlighting the need for data governance, informed consent practices, and transparent algorithmic accountability. Ensuring robust data protection protocols and user consent frameworks is critical for maintaining trust and encouraging the safe use of AI in skincare personalization. Companies must adhere to stringent data privacy regulations, such as the General Data Protection Regulation (GDPR), and implement strong cybersecurity measures to protect user information.

In addition to privacy concerns, another limitation of AI is the potential for algorithmic bias. AI models are trained on large datasets, and if these datasets lack diversity, the resulting skincare recommendations may not be equally effective across all populations [34]. For example, there is a theoretical concern that an AI system trained predominantly on datasets featuring lighter skin tones could generate suboptimal, or in some cases, inappropriate, skincare recommendations for individuals with darker skin tones. This potential bias stems from inadequate representation in training data, which may limit the algorithm’s ability to accurately assess conditions such as hyperpigmentation, acne, or sensitivity responses that present differently across diverse skin types. As a result, the effectiveness of AI-based skincare recommendations may vary significantly across ethnic groups, age ranges, and skin types, perpetuating disparities in both outcomes and user trust [35]. Addressing this issue requires building more inclusive datasets representing a comprehensive range of skin tones, types, and conditions. Moreover, ongoing validation and auditing of AI algorithms are necessary to detect and correct biases. While AI excels in delivering scientific precision, it may not always account for subjective factors like personal preferences for product texture, scent, or overall user experience, which remain crucial elements in skincare choices. Thus, human input and dermatological expertise remain important in balancing scientific recommendations with individual preferences. Additionally, technological limitations, such as the accuracy of skin analysis from smartphone cameras, can affect the reliability of AI recommendations.

## Conclusions

AI is transforming the skincare industry by offering personalized, data-driven solutions that adapt to the unique and evolving needs of each individual. By analyzing large volumes of data, including intrinsic factors like genetics and skin type, as well as extrinsic influences such as environmental exposure and lifestyle, AI has the potential to generate more personalized and data-informed skincare recommendations. While still evolving, these systems show promise in improving regimen precision compared to traditional one-size-fits-all approaches. This shift from traditional, generalized approaches to more targeted, responsive solutions enhances skin health and improves user satisfaction through more effective, real-time adjustments. Nonetheless, challenges like data privacy concerns, algorithmic bias, and technological limitations need to be addressed to ensure the fair and safe application of AI technology. Companies must prioritize data security and work toward eliminating biases in AI models to make personalized skincare accessible and effective for all users. Moreover, while AI excels in delivering precise, science-based recommendations, the human touch remains essential, particularly in accounting for personal preferences and subjective skincare experiences. As AI continues to evolve, its role in skincare will likely expand, offering more advanced, inclusive, and efficient ways to achieve optimal dermatological outcomes. Future advancements may include integrating more sophisticated sensors, improving the accuracy of AI algorithms, and enhancing the user experience by incorporating personal preferences. With continued advancements in AI technology and improvements in data protection and inclusivity, the future of skincare personalization looks promising, potentially revolutionizing how individuals approach skin health.
